# Mcl-1 Differentially Regulates Autophagy in Response to Changes in Energy Status and Mitochondrial Damage

**DOI:** 10.3390/cells11091469

**Published:** 2022-04-27

**Authors:** Alexandra G. Moyzis, Navraj S. Lally, Wenjing Liang, Rita H. Najor, Åsa B. Gustafsson

**Affiliations:** Skaggs School of Pharmacy and Pharmaceutical Sciences, University of California San Diego, La Jolla, CA 92130, USA; amoyzis@gmail.com (A.G.M.); nlally@ucsd.edu (N.S.L.); wel257@health.ucsd.edu (W.L.); rnajor@health.ucsd.edu (R.H.N.)

**Keywords:** *Mcl-1*, Bnip3, mitochondria, autophagy, mitophagy, heart

## Abstract

Myeloid cell leukemia-1 (*Mcl-1*) is a unique antiapoptotic Bcl-2 member that is critical for mitochondrial homeostasis. Recent studies have demonstrated that *Mcl-1*′s functions extend beyond its traditional role in preventing apoptotic cell death. Specifically, data suggest that *Mcl-1* plays a regulatory role in autophagy, an essential degradation pathway involved in recycling and eliminating dysfunctional organelles. Here, we investigated whether *Mcl-1* regulates autophagy in the heart. We found that cardiac-specific overexpression of *Mcl-1* had little effect on baseline autophagic activity but strongly suppressed starvation-induced autophagy. In contrast, *Mcl-1* did not inhibit activation of autophagy during myocardial infarction or mitochondrial depolarization. Instead, overexpression of *Mcl-1* increased the clearance of depolarized mitochondria by mitophagy independent of Parkin. The increase in mitophagy was partially mediated via *Mcl-1*′s LC3-interacting regions and mutation of these sites significantly reduced *Mcl-1*-mediated mitochondrial clearance. We also found that *Mcl-1* interacted with the mitophagy receptor Bnip3 and that the interaction was increased in response to mitochondrial stress. Overall, these findings suggest that *Mcl-1* suppresses nonselective autophagy during nutrient limiting conditions, whereas it enhances selective autophagy of dysfunctional mitochondria by functioning as a mitophagy receptor.

## 1. Introduction

Autophagy is an essential degradation pathway that is necessary for cellular homeostasis. When nutrients are limited, autophagic degradation is nonselective, whereby cytoplasmic components are engulfed in autophagosomes and targeted to the lumen of the lysosome [1]. In this recycling process, degradation of the cargo by lysosomal proteases liberates the basic building blocks of proteins, lipids, and organelles to fuel the synthesis of new macromolecules. Autophagy also functions as a critical cellular quality control mechanism and is responsible for selectively eliminating cytotoxic protein aggregates and dysfunctional organelles. Specifically, the selective removal of mitochondria by autophagosomes is known as mitochondrial autophagy (mitophagy) [2]. Beyond the generation of ATP through oxidative phosphorylation, mitochondria have key functions ranging from regulating programmed cell death to heme synthesis [3]. However, dysfunctional mitochondria can become excessive generators of reactive oxygen species and activators of cell death. Therefore, mitophagy is a critical and tightly regulated process, as either failure to remove damaged mitochondria or the unnecessary degradation of functional mitochondria can be detrimental to cells.

As nonselective autophagy is activated during starvation to maintain cellular energy levels, a mechanism likely exists to safeguard healthy mitochondria against autophagic degradation under these conditions. Indeed, an in vitro proteomics study reported that the autophagic response to nutrient deprivation is ordered; that is, cytoplasmic proteins and nonessential components are degraded early on, followed by different organelles at later time points [4]. However, how mitochondria and other organelles are protected from degradation during nonselective autophagy remains unknown. 

Mitophagy predominantly occurs through two distinct pathways. The first and most extensively studied pathway is PINK1/Parkin-mediated mitophagy, where Parkin is recruited to depolarized mitochondria by PINK1. At the mitochondria, Parkin ubiquitinates numerous proteins on the outer membrane, which induces the recruitment of autophagy adaptor proteins such as p62 [2]. The adaptor proteins bind to ubiquitinated proteins on the mitochondria and to LC3/GABARAP proteins on the autophagosomal membrane to effectively tether the mitochondrion to the autophagosome. Mitophagy can also be induced independently of Parkin by specialized proteins called mitophagy receptors that are anchored in the outer mitochondrial membrane [2]. These mitophagy receptors possess an LC3-interacting region (LIR) through which they directly bind to LC3/GABARAP on the autophagosome [5]. While the mitophagy receptors have structural homology and overlapping functions, they are involved in regulating mitophagy under distinct conditions. For example, the mitophagy receptors Bnip3 and Fundc1 are largely responsible for mitochondrial degradation during hypoxia and mitochondrial depolarization [6,7], while Nix is essential for mitochondrial clearance during reticulocyte development [8,9]. 

Moreover, the antiapoptotic Bcl-2 family members are known to inhibit permeabilization of the outer mitochondrial membrane, but studies have reported that they can also suppress autophagy by sequestering the core autophagy protein Beclin1 and preventing it from initiating autophagosome formation [10]. Myeloid cell leukemia-1 (*Mcl-1*) is an atypical prosurvival Bcl-2 family member that is localized to both the outer mitochondrial membrane and the matrix, where it has unique functions in maintaining mitochondrial homeostasis [11,12,13,14,15]. However, its function in regulating autophagy is unclear as current studies are conflicting. For instance, loss of *Mcl-1* in mouse cortical neurons leads to increased activation of autophagy [12], while deletion of *Mcl-1* in the heart leads to a disruption in autophagosome formation [15]. *Mcl-1* has also been reported to inhibit Parkin-mediated mitophagy [16], yet it can promote mitochondrial clearance by functioning as a mitophagy receptor [17]. Given these conflicting reports, how *Mcl-1* differentially regulates autophagy and mitophagy needs to be further investigated.

*Mcl-1* is essential for cardiac homeostasis, and deletion of *Mcl-1* in myocytes leads to the rapid development of heart failure despite little activation of apoptosis [15,18], demonstrating the importance of its other functions. Here, we explore how *Mcl-1* regulates autophagy and mitophagy in cells and the heart. We report that *Mcl-1* in the outer mitochondrial membrane inhibits nonselective autophagy activated by Rapamycin or nutrient deprivation but not selective autophagy involved in cellular quality control. Instead, *Mcl-1* promotes mitophagy of depolarized mitochondria independent of both Parkin and its traditional antiapoptotic function. Thus, our findings suggest that as a mitochondrial-localized protein, *Mcl-1* might function to protect functional mitochondria from nonselective autophagic degradation while promoting the clearance of damaged mitochondria. 

## 2. Materials and Methods

### 2.1. Cell Culture and Treatments

Immortalized mouse embryonic fibroblasts (MEFs) were maintained at 37 °C in a 5% CO_2_ atmosphere and cultured in media composed of high glucose DMEM (Thermo Fisher Scientific, Waltham, MA, USA, 10569-010) supplemented with 10% fetal bovine serum (Thermo Fisher Scientific, 16000-044), 100 U mL^−1^ penicillin and 100 mg mL^−1^ streptomycin (Gemini, 400-109). For treatments, cell culture media was supplemented with 25 μM carbonyl cyanide 4-(trifluoromethoxy) phenylhydrazone (FCCP) (Sigma-Aldrich, St. Louis, MO, USA, C2920), 50 nM Bafilomycin A1 (BafA1) (Sigma, 19-148), or 100 nM Rapamycin (LC Laboratories, Woburn, MA, USA, R-5000) for the indicated amounts of time. For hypoxia experiments, cells were placed in cell culture media supplemented with 40 mM HEPES (pH 7.4) (Thermo Fisher Scientific, 15630-080) and incubated in hypoxic pouches (GasPak EZ, BD Biosciences, Franklin Lakes, NJ, USA, 260683) equilibrated to 95% N_2_, 5% CO_2_.

### 2.2. cDNA Constructs, Transient Transfections, and Adenoviral Infections

The *Mcl-1* cDNA has been previously described and was provided by Dr. Joseph Opferman from St. Jude Children’s Research Hospital [11]. The *Mcl-1*-LIR1-mutant (F227A, V230A) and *Mcl-1*-LIR3-mutant (F299A, V302A) were generated by PCR-based site-directed mutagenesis using the *Mcl-1* construct as the template. The *Mcl-1*-LIR2-mutant (W242A, I245A) and *Mcl-1*-LIR123-mutant (F227A, V230A, W242A, I245A, F229A, V302A) were synthesized by GenScript. The GFP-LC3, Myc-Bnip3, and *Mcl-1*-BH3-mutant constructs have been previously described [14,19,20].

Cells were transiently transfected with cDNA constructs using FuGENE 6 Transfection reagent (Promega, Madison, WI, USA, E2691) according to the manufacturer’s instructions. For adenoviral infections, cells were cultured in DMEM + 2% heat-inactivated serum (Gibco, A3840001) containing the indicated viruses for 3 h and then rescued with cell culture media.

### 2.3. Western Blot Analysis, Coimmunoprecipitation, and Subcellular Fractionation

MEFs were lysed in ice-cold lysis buffer containing 50 mM Tris-HCl, 150 mM NaCl, 1 mM EGTA, 1 mM EDTA, 1% Triton X-100 and cOmplete Protease Inhibitor Cocktail (Roche, Basel, Switzerland, 11697498001), and lysates were passed through a 27G needle. Tissue samples were minced in detergent-free lysis buffer and then homogenized by Polytron. Triton X-100 was then added to homogenates to a final concentration of 1%. Resulting cell and tissue lysates were incubated on ice for 45 min and then cleared by centrifugation at 20,000× *g* for 20 min at 4 °C.

To isolate mitochondria, tissue was minced in ice-cold fractionation buffer (250 mM sucrose, 5 mM KH_2_PO_4_, 2 mM MgCl_2_, 10 mM MOPS, pH 7.4, 1 mM EGTA, 0.1% fatty acid-free BSA, and protease inhibitor cocktail), homogenized at low speed by Polytron, and further homogenized using the Potter-Elvehjem Teflon tissue grinder (Sigma, P7734). Resulting homogenates were centrifuged at 600× *g* for 5 min at 4 °C to remove unbroken material, and the supernatant was further centrifuged at 6000× *g* at 4 °C to separate the mitochondrial fraction from the cytosol. The mitochondrial pellet was resuspended in fractionation buffer and centrifuged at 6000× *g* for 10 min at 4 °C to wash the fraction. The final pellet was resuspended in lysis buffer. Protein concentrations were determined using Bradford assay, and lysates were supplemented with 1X sample buffer (Invitrogen, Waltham, MA, USA, NP0008) and 50 mM dithiothreitol (DTT). 

Immunoprecipitation experiments were performed as previously described [19]. Briefly, cell lysates were precleared with Protein A-agarose (Santa Cruz Biotechnology, Inc., Dallas, TX, USA, sc-2001) for 30 min and incubated with an anti-Myc antibody (Sigma, M4439) overnight. Antibody-bound protein complexes were captured with Protein A-agarose for 2 h, washed with 1X calcium chloride and magnesium chloride-free phosphate-buffered saline (PBS), and eluted in 2X sample buffer (Invitrogen, NP0007) and 50mM DTT.

Proteins were separated on Invitrogen NuPAGE Bis-tris gels and transferred to nitrocellulose membranes. Membranes were probed with the following antibodies in 5% milk: Actin (1:1000, GeneTex, GTX11003), Beclin1 (1:1000, Santa Cruz, sc-11427), Bnip3 (1:500, Sigma, B7931), Gapdh (1:1000, GeneTex, GTX627408), LC3 (1:1000, Cell Signaling, 4108), *Mcl-1* (1:1000, Rockland, 600-401-394), p62 (1:1000, Abcam, ab56416), Tim23 (1:1000, BD Biosciences, 611222), and Tubulin (1:1000, Sigma, T6074). Membranes were washed with 0.1% TBST, probed with goat anti-rabbit (Invitrogen, 32460) or goat anti-mouse (Invitrogen, 62-6520) HRP-conjugated secondary antibodies, and imaged using a ChemiDoc XRS+ System (Bio-Rad). Densitometry was performed using Image Lab 4.1 software (Bio-Rad).

### 2.4. Immunofluorescence

Cells were fixed in 4% paraformaldehyde at 37 °C for 20 min, permeabilized with 0.2% Triton X-100 in 1X PBS at 37 °C for 30 min, and incubated with blocking buffer consisting of 5% normal goat serum (Vector Labs, S-1000-20). Cells were stained with antibodies against Cytochrome c (1:100, BD Pharmingen #556432) or Tom20 (1:100, Santa Cruz, sc-11415) overnight at 4 °C. Cells were then rinsed with 1X PBS and incubated with an Alexa Fluor 594 secondary antibody (1:200, Invitrogen, A11037) for 1 h at 37 °C. Lastly, cells were stained with Hoechst 33342 (1:1000, Invitrogen, H3570) to label nuclei.

Fluorescence images were captured using a Nikon Eclipse Ti2-E with a motorized XYZ-stage fitted with a Plan-Apochromat lambda 60X NA 1.40 oil immersion objective. Z stacks were separated by 0.3 mm and acquired with a DS-Qi2 camera (Nikon Instruments, Melville, NY, USA) illuminated by a solid-state white light excitation source (Lumencor, Beaverton, OR, USA). Images were processed by deconvolution and compressed into extended depth-of-focus (EDF) images by NIS-Elements AR GA3 software. To evaluate mitochondrial clearance, 200 cells were counted per condition in each experiment. To assess GFP-LC3 and mitochondrial colocalization, the number of GFP-LC3 puncta overlapping with mitochondria was manually scored. A minimum of 20 cells were scored per condition in each experiment.

### 2.5. Mouse Experiments

All animal experiments were carried out in accordance with institutional guidelines and approved by the Institutional Animal Care and Use Committee at UCSD. The αMHC-*Mcl-1*_OM_ transgenic mice have been previously described [14]. Age- and sex-matched 8–12-week-old mice were used for all experiments, and data represent results obtained from both sexes. Fasting was initiated by removing the chow for up to 24 h. To induce myocardial infarction, adult mice were subjected to permanent ligation of the left anterior descending coronary artery (LAD) as previously described [21]. Briefly, mice were anesthetized with isoflurane, intubated, and ventilated. The LAD was then ligated with an 8-0 silk suture, and the chest cavity was immediately closed with sutures and tissue adhesive. For experiments measuring in vivo autophagic flux, saline (vehicle) or 80 mg/kg of chloroquine (Sigma, C6628) was administered intraperitoneally (IP).

### 2.6. Statistical Analysis

All values are expressed as mean ± standard error of mean (SEM). Student’s *t*-test was used to evaluate differences between 2 sets of data. ANOVA, followed by Dunnett’s or Bonferroni’s multiple comparison test, was used for data comparison of more than 2 groups using GraphPad Software (GraphPad Software, Inc., San Diego, CA, USA). Values of *p* < 0.05 were considered statistically significant. Details regarding the n for each experiment can be found in the figure legends.

## 3. Results

**Figure 1:**
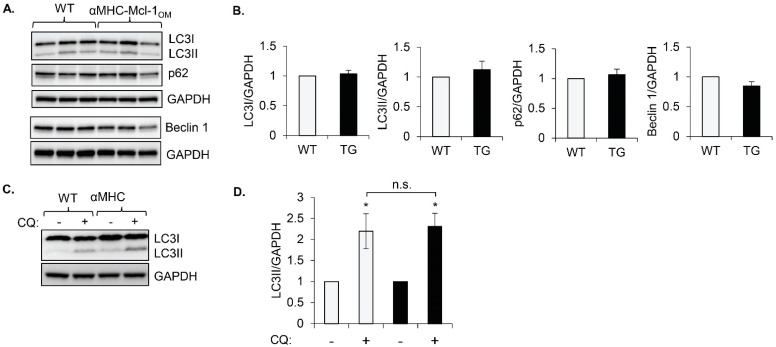
Baseline autophagic activity in hearts is not inhibited by *Mcl-1*_OM_ overexpression. (**A**) Representative Western blots of LC3, p62, and Beclin1 protein levels in hearts of WT and αMHC-*Mcl-1*_OM_ mice. GAPDH was used as a loading control. (**B**) Quantification of LC3I, LC3II, p62, and Beclin1 protein levels (*n* = 3–5). (**C**) Representative Western blots of LC3 and GAPDH protein levels in hearts of WT and αMHC-*Mcl-1*_OM_ mice after injection with vehicle (−) or 80 mg/kg chloroquine (CQ) (+) for 2 h. (**D**) Quantification of LC3II levels (*n* = 4–5, * *p* < 0.05 vs. CQ (−); n.s., not significant). WT = wild type, TG = transgenic. Data are mean ± SEM. Statistical tests: Student’s t-test or ANOVA, followed by Bonferroni’s multiple comparison test.

To investigate if *Mcl-1* regulates autophagy in the heart, we utilized mice with cardiac-specific overexpression of *Mcl-1* that is specifically targeted to the outer mitochondrial membrane (αMHC-*Mcl-1*_OM_). We have previously reported that increased levels of *Mcl-1*_OM_ in hearts have little effect on cardiac structure or function under baseline conditions [14]. To assess baseline autophagic activity, we monitored levels of key autophagy markers LC3, p62, and Beclin1. Conversion of cytosolic LC3I to autophagosome-bound LC3II correlates with an increase in autophagosome formation [22], while the adaptor protein p62 accumulates in cells with decreased autophagic activity [23]. Beclin1 is an important upstream regulator that is involved in initiating autophagosome formation [24]. We found no significant changes in the levels of the autophagy proteins LC3I, LC3II, p62, and Beclin1 in hearts overexpressing *Mcl-1*_OM_ under baseline conditions (Figure 1A,B). To further confirm that *Mcl-1*_OM_ has little effect on autophagy at baseline, we assessed autophagic flux by injecting WT and αMHC-*Mcl-1*_OM_ mice with chloroquine, which inhibits lysosomal acidification and autophagic degradation resulting in an accumulation of LC3II [15]. Wild-type and *Mcl-1*_OM_ transgenic mouse hearts showed a similar increase in LC3II levels after chloroquine injections, indicating that autophagic flux was intact in hearts overexpressing *Mcl-1*_OM_ (Figure 1C,D). These results suggest that there are no inherent effects of *Mcl-1*_OM_ overexpression in the heart on autophagic activity at baseline.

**Figure 2:**
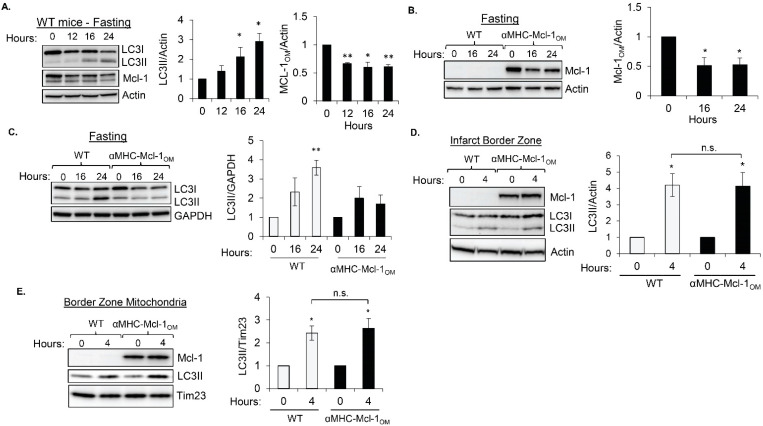
Overexpression of *Mcl-1*_OM_ prevents induction of autophagy in hearts during fasting but not in response to myocardial infarction. (**A**) Representative Western blot and quantification of endogenous *Mcl-1* and LC3II protein levels in the hearts of wild-type (WT) mice after 0, 12, 16, and 24 h of fasting (*n* = 3). (**B**) Representative Western blot and quantification of *Mcl-1*_OM_ transgene levels in hearts after 0, 16, and 24 h of fasting (*n* = 4). (**C**) Representative Western blot and quantification of LC3II levels in hearts of WT and αMHC-*Mcl-1*_OM_ mice after 0, 16, and 24 h of fasting (*n* = 4). (**D**) Representative Western blot and quantification of LC3II protein levels in the infarct border zone 4 h postmyocardial infarction (*n* = 5). (**E**) Representative Western blot and quantification of LC3II protein levels in mitochondrial fractions isolated from the infarct border zone of hearts in WT and αMHC-*Mcl-1*_OM_ mice at 4 h after myocardial infarction (*n* = 5). * *p* < 0.05 and ** *p* < 0.01 compared to 0 h control, n.s., not significant. Data are mean ± SEM. Statistical test: ANOVA, followed by Dunnett’s or Bonferroni’s multiple comparison test.

Next, we investigated if *Mcl-1* affects induction of autophagy in response to various challenges. Under baseline conditions, autophagy is primarily involved in quality control by eliminating cellular waste, such as protein aggregates and dysfunctional or aged organelles. In contrast, induction of autophagy in response to nutrient deprivation functions to provide the cells with substrates for energy metabolism and amino acids for protein synthesis by recycling cytosolic cargo [1]. First, we examined the relationship between *Mcl-1* levels and autophagosome formation in the hearts of mice that had been subjected to fasting. We found that fasting of mice for up to 24 h led to a decrease in endogenous *Mcl-1*_OM_ levels in the heart, which correlated with an increase in autophagosome formation as measured by increased LC3II levels (Figure 2A). Since the antiapoptotic Bcl-2 proteins have been reported to inhibit autophagy, our findings suggested that *Mcl-1* degradation might allow for autophagy to proceed. To further investigate whether *Mcl-1*_OM_ levels regulate the induction of autophagy in hearts during fasting, WT and αMHC-*Mcl-1*_OM_ mice were subjected to fasting for 0, 16, and 24 h. Similar to endogenous *Mcl-1*_OM_ in WT mouse hearts, *Mcl-1*_OM_ protein from the transgene was significantly reduced in response to fasting at 16 and 24 h (Figure 2B). Despite this decrease in *Mcl-1*_OM_ levels, the protein was still maintained at levels that were noticeably higher than endogenous *Mcl-1*_OM_. Moreover, while LC3II levels significantly increased in the hearts of WT mice after 24 h of fasting, hearts overexpressing *Mcl-1*_OM_ failed to increase LC3II at the same time point (Figure 2C). These findings suggest that although *Mcl-1* has little effect on autophagy at baseline, it inhibits the induction of autophagy under nutrient-limited conditions. 

While induction of autophagy during nutrient deprivation is associated with nonselective degradation and recycling, stress-induced autophagy is primarily responsible for the selective removal of damaged and potentially cytotoxic material in the cell. Therefore, we investigated the effect of *Mcl-1*_OM_ overexpression in hearts on the induction of autophagy in response to myocardial infarction, a stress that is associated with increased mitochondrial damage and activation of autophagy in the border zone of the infarct [21,25]. WT and αMHC-*Mcl-1*_OM_ TG mice were subjected to myocardial infarction by permanent ligation of the left anterior descending artery (LAD) and induction of autophagy was assessed in the infarct border zone by Western blotting for LC3. In contrast to fasting, we found a similar increase in LC3II levels in the border zone in both WT and αMHC-*Mcl-1*_OM_ mice, despite high levels of *Mcl-1*_OM_ (Figure 2D). This suggested that *Mcl-1* did not inhibit autophagy under these conditions. Additionally, mitochondrial damage associated with myocardial infarction leads to increased mitophagy in the infarct border zone [21]. Because *Mcl-1* localizes to mitochondria, we investigated whether *Mcl-1*_OM_ suppressed mitophagy under these conditions by evaluating levels of the autophagy protein LC3II associated with mitochondria as an indicator of mitophagy. However, Western blot analysis of mitochondrial fractions prepared from the border zone tissue confirmed a similar increase in LC3II in mitochondrial fractions in the hearts of WT and αMHC-*Mcl-1*_OM_ mice (Figure 2E). Furthermore, *Mcl-1*_OM_ levels remained unchanged in the border zone at this time point, suggesting that autophagy and mitophagy are intact despite elevated levels of *Mcl-1* in the outer mitochondrial membrane. 

**Figure 3:**
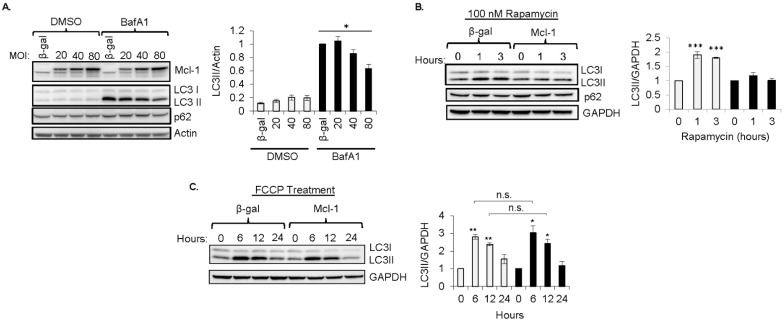
*Mcl-1* overexpression inhibits induction of nonselective, but not selective, autophagy. (**A**) LC3II levels accumulate in cells overexpressing β-gal or *Mcl-1* (at 20, 40, or 80 multiples of infection (MOI)) in the presence of 50 nM Bafilomycin A1 for 4 h (*n* = 4). (**B**) Representative blot and quantification of LCII levels in MEFs treated with 100 nM Rapamycin for 0, 1, or 3 h (*n* = 3). (**C**) Representative Western blot and quantification of LC3II levels in MEFs in response to 0, 6, 12, or 24 h of treatment with FCCP (25 μM, *n* = 3). * *p* < 0.05, ** *p* < 0.01, *** *p* < 0.0001 vs. 0 h. n.s., not significant. Data are mean ± SEM. Statistical test: ANOVA, followed by Dunnett’s or Bonferroni’s multiple comparison test.

To further characterize the specific conditions under which *Mcl-1* inhibits the induction of autophagy, we investigated the effect of *Mcl-1* overexpression on autophagy in mouse embryonic fibroblasts (MEFs) at baseline or in response to various treatments. To assess whether *Mcl-1* inhibited baseline autophagy, autophagosome formation was evaluated in cells overexpressing β-gal or *Mcl-1* in the presence of Bafilomycin A1, an inhibitor of the vacuolar-type H+-ATPase (V-ATPase) that prevents autophagic degradation [26]. Similar to our findings in αMHC-*Mcl-1*_OM_ transgenic mouse hearts, overexpression of *Mcl-1* had little effect on autophagosome formation at baseline except at the highest concentration of 80 MOI (Figure 3A). Next, we investigated the effect of *Mcl-1* overexpression on the pharmacological activation of autophagy by Rapamycin, an inhibitor of mTOR and a potent activator of autophagy [27]. We found that overexpression of *Mcl-1* suppressed Rapamycin-stimulated autophagosome formation in MEFs (Figure 3B). Next, we examined if *Mcl-1* affected activation of autophagy in response to FCCP, a mitochondrial uncoupler and potent inducer of mitophagy [28]. Interestingly, *Mcl-1* overexpression did not affect autophagosome formation in response to FCCP treatment (Figure 3C). These findings suggest that *Mcl-1* limits the induction of nonselective autophagy by Rapamycin in the absence of cellular damage but does not inhibit activation of selective autophagy that is involved in cellular quality control.

**Figure 4:**
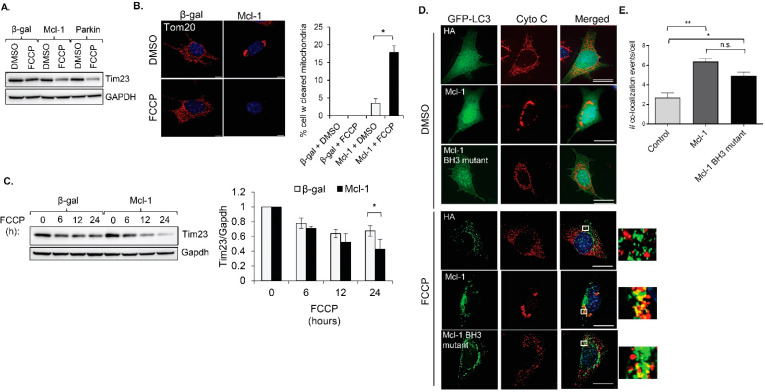
*Mcl-1* overexpression enhances FCCP-mediated mitophagy. (**A**) Representative Western blot of Tim23 in MEFs infected with β-gal, *Mcl-1*, or Parkin prior to treatment with DMSO or FCCP (25 μM, 24 h). (**B**) Representative images of MEFs infected with β-gal or *Mcl-1*. Cells were treated with DMSO or FCCP (25 μM, 12 h), fixed, and stained with anti-Tom20. Quantification of cells with cleared mitochondria (*n* = 3, 200 cells were analyzed per experiment). Scale bar = 10 μm. (**C**) Representative blot and quantification of Tim23 protein levels in an FCCP (25 μM) time-course experiment (*n* = 3). (**D**) Representative images of GFP-LC3 and Cytochrome c in cells. (**E**) quantification of GFP-LC3 and mitochondrial colocalization events per cell (*n* = 3, 18–20 cells were analyzed per experiment). * *p* < 0.05, ** *p* < 0.01, n.s., not significant. Data are mean ± SEM. Scale bar = 20 μm. Statistical test: ANOVA, followed by Bonferroni’s multiple comparison test.

The finding that *Mcl-1* failed to inhibit autophagy in response to myocardial infarction and FCCP treatment prompted us to investigate the effect of *Mcl-1* on mitochondrial clearance. Parkin is an important E3 ubiquitin ligase involved in labeling depolarized mitochondria for mitophagy [2]. Since MEFs do not have detectable levels of Parkin [28], they are relatively inefficient at clearing their mitochondria in response to stress. To investigate the effect of *Mcl-1* on mitophagy, MEFs were infected with adenoviruses encoding β-gal, *Mcl-1*, or Parkin prior to treatment with FCCP to induce mitophagy. Mitochondrial clearance was assessed by monitoring levels of the mitochondrial protein Tim23 and by staining cells for the mitochondrial marker Tom20. As expected, overexpression of Parkin led to enhanced mitochondrial clearance after FCCP treatment (Figure 4A). As previously reported, overexpression of *Mcl-1* alone led to increased mitochondrial fission and perinuclear nuclear aggregation in cells [14] (Figure 4B,D). Interestingly, *Mcl-1* overexpression also led to increased clearance of mitochondria after treatment with FCCP compared to cells overexpressing β-gal (Figure 4A–C). In addition, fluorescence analysis of cells confirmed a significant increase in the colocalization between GFP-LC3-positive autophagosomes and mitochondria in cells overexpressing *Mcl-1* compared to β-gal (Figure 4D,E). To investigate the connection between *Mcl-1*′s effect on mitophagy and its antiapoptotic function, we overexpressed an *Mcl-1* construct with mutations in the BH3 domain that disrupt its antiapoptotic function [14] and assessed its effect on mitophagy. The *Mcl-1* BH3 mutant also increased colocalization between autophagosomes and mitochondria in response to FCCP treatment (Figure 4D,E). These results suggest that *Mcl-1* is involved in facilitating the clearance of depolarized mitochondria and that a functional BH3 domain is not necessary. 

**Figure 5:**
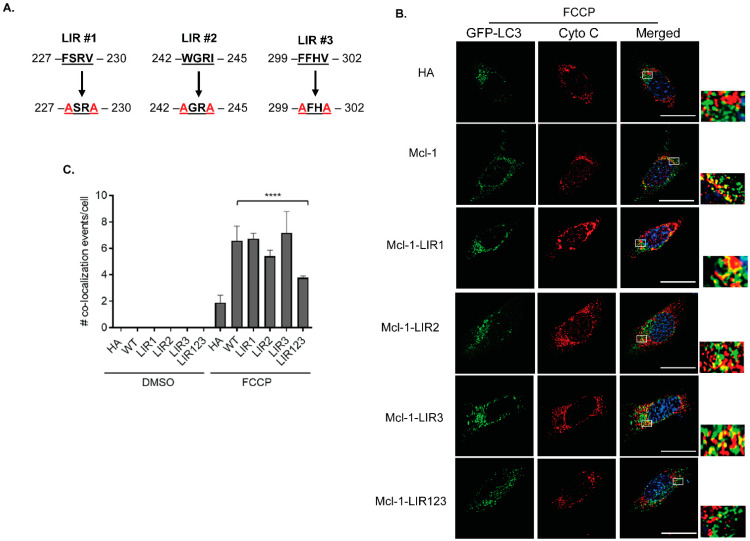
*Mcl-1*-mediated mitophagy is partially dependent on its LC3-interacting region (LIR) motifs. (**A**) Mutation of *Mcl-1*’s putative LIR motifs. (**B**) Representative fluorescence images of GFP-LC3 and Cytochrome c colocalization in cells overexpressing HA, *Mcl-1*, or *Mcl-1* LIR mutants. (**C**) Quantification of colocalization events between autophagosomes and mitochondria after treatment with 25 μM FCCP (*n* = 3–6, 18–20 cells were scored in each experiment). **** *p* < 0.0001. Data are mean ± SEM. Scale bar = 20 μm. Statistical test: ANOVA, followed by Bonferroni’s multiple comparison test.

While Parkin uses ubiquitin to label mitochondria for autophagy, mitophagy receptors integrated with the outer mitochondrial membrane can facilitate mitochondrial clearance by directly interacting with LC3 on autophagosomes through their conserved LC3-interacting region (LIR) motifs [29,30]. *Mcl-1* is also anchored in the outer mitochondrial membrane via a transmembrane domain and contains three canonical LIR motifs (W/F/Y-X-X-L/I/V) in its C-terminal region (Figure 5A). To investigate the functions of these LIR motifs in facilitating mitophagy in MEFs, we generated four different *Mcl-1* mutants with mutations in one or all of the different LIR motifs; LIR#1: *Mcl-1* (F227A, V230A), LIR#2: *Mcl-1* (W242A, I245A), LIR#3: *Mcl-1* (F299A, V302A), and LIR#123: *Mcl-1* (F227A, V230A, W242A, I245A, F299A, V302A). Mitophagy was evaluated by assessing colocalization between mitochondria and LC3-positive autophagosomes in MEFs overexpressing GFP-LC3 plus wild-type *Mcl-1* or the various LIR mutants. We found that mutating the individual LIR motifs had little effect on the colocalization between GFP-LC3-positive autophagosomes and mitochondria (Figure 5B). Only simultaneous mutation of all 3 LIR motifs in *Mcl-1* led to a significant reduction in the colocalization between LC3 puncta and mitochondria (Figure 5B,C), confirming that *Mcl-1* can function as a mitophagy receptor to directly facilitate their clearance.

**Figure 6:**
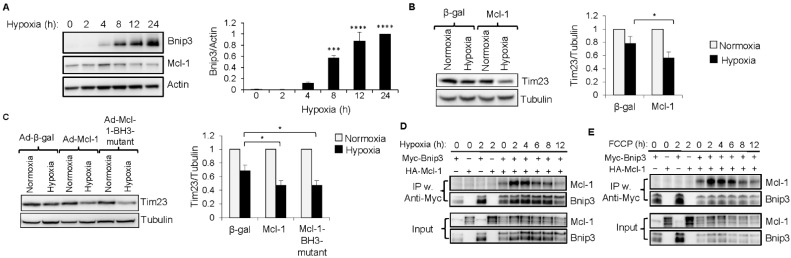
*Mcl-1* overexpression enhances hypoxia-mediated mitophagy. (**A**) Representative blot of endogenous Bnip3 and *Mcl-1* and quantification of Bnip3 in MEFs exposed to hypoxia for up to 24 h (*n* = 3). (**B**) Representative Western blot and quantification of Tim23 in normoxic and hypoxic cells overexpressing either β-gal or *Mcl-1* (*n* = 3). (**C**) Representative Western blot and quantification of Tim23 in normoxic and hypoxic cells overexpressing β-gal, *Mcl-1*, or the *Mcl-1* BH3-mutant (*n* = 3). (**D**) Coimmunoprecipitation of Bnip3 using anti-Myc in MEFs overexpressing Bnip3 and *Mcl-1*, followed by Western blotting for Bnip3 and *Mcl-1* in the IP and cell lysates (input). Cells were cultured under hypoxic conditions for the indicated time points. (**E**) Coimmunoprecipitation (IP) of Bnip3 using anti-Myc in MEFs overexpressing Bnip3 and *Mcl-1*, followed by Western blotting for Bnip3 and *Mcl-1* in the IP and total cell lysates (input). Cells were treated with 25 μM FCCP for the indicated time points. * *p* < 0.05, *** *p* < 0.001, **** *p* < 0.0001. Data are mean ± SEM. Statistical test: ANOVA, followed by Dunnett’s or Bonferroni’s multiple comparison test.

Since mitophagy was not completely abrogated in cells overexpressing the *Mcl-1* LIR#123 mutant, it suggested that *Mcl-1* might also be regulating mitophagy via an additional mechanism. Therefore, we investigated if *Mcl-1* coordinates with other mitophagy receptors to ensure removal of dysfunctional mitochondria. The atypical BH3-only protein Bnip3 functions as a mitophagy receptor under certain conditions [19] and is strongly induced by hypoxia [31] (Figure 6A). Similar to observation with FCCP treatment, we found that overexpression of *Mcl-1* enhanced hypoxia-induced mitochondrial clearance in cells exposed to hypoxia (Figure 6B). The *Mcl-1*-BH3 mutant was also effective at enhancing mitophagy in response to hypoxia (Figure 6C), further confirming that a functional BH3 domain in *Mcl-1* is not necessary to facilitate mitophagy. Finally, coimmunoprecipitation studies demonstrated an increase in the interaction between Bnip3 and *Mcl-1* as early as 2 h after initiating hypoxia (Figure 6D). Similarly, there was an increase in the interaction between *Mcl-1* and Bnip3 in response to FCCP treatment (Figure 6E). Overall, this suggests that *Mcl-1* might also be facilitating mitophagy via its interaction with Bnip3. 

## 4. Discussion

**Figure 7:**
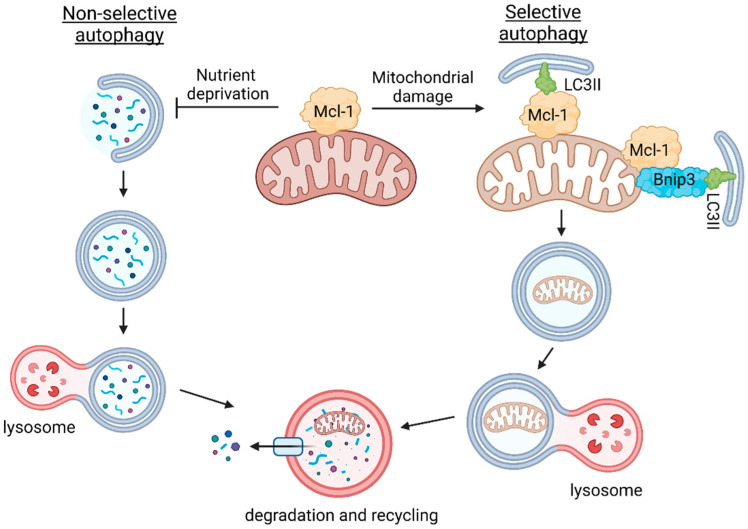
Opposing effects of *Mcl-1* on nonselective autophagy and selective mitochondrial autophagy (mitophagy). *Mcl-1* at the mitochondria suppresses formation of autophagosomes during nutrient deprivation possibly to protect healthy mitochondria from degradation. In contrast, *Mcl-1* promotes autophagy of dysfunctional mitochondria by functioning as a mitophagy receptor or by coordinating with Bnip3. Created with BioRender.com (accessed on 21 March 2022).

*Mcl-1* has been implicated in regulating both autophagy and mitophagy, but its exact functional role remains unclear due to conflicting reports. Here, we identified distinct functions for *Mcl-1* in regulating autophagy and mitophagy that are independent of its antiapoptotic function. First, we show that while *Mcl-1* strongly suppresses activation of autophagy in response to nutrient deprivation, it has little effect on baseline autophagy or mitochondrial stress-induced autophagy. During increased stress, *Mcl-1* instead promotes the degradation of depolarized mitochondria by functioning as a mitophagy receptor and possibly by interacting with Bnip3. Therefore, the distinction lies in the fact that *Mcl-1* inhibits nonselective autophagy involved in general recycling but positively regulates selective autophagy involved in cellular quality control. Overall, these findings suggest that *Mcl-1* might protect cells by shielding healthy mitochondria from nonselective autophagy when nutrients are limited while enhancing the clearance of harmful dysfunctional mitochondria in response to stress (Figure 7). These functions are consistent with *Mcl-1*’s role as a prosurvival protein. 

It is well established that starvation leads to activation of nonselective autophagy in an effort to maintain energy homeostasis [32]. Our data clearly demonstrate that overexpression of *Mcl-1* suppressed activation of autophagy in response to nutrient deprivation downstream of mTOR. Because of its mitochondrial localization, it is likely that *Mcl-1*’s primary function is to inhibit autophagosome formation near mitochondria to protect them from degradation during nutrient limiting conditions. Mitochondria are the primary generators of ATP in the heart [3], and unnecessary degradation of functional mitochondria by nonselective autophagy during nutrient limiting conditions would further contribute to the energy deficiency. Consistent with our findings, a proteomics analysis of amino-acid-starved cells revealed that while cytosolic proteins are rapidly degraded, organelles are not degraded until later time points [4]. Thus, *Mcl-1* may be involved in the signaling mechanisms that allow mitochondria to evade degradation via nonselective autophagy in the initial phase of bulk degradation. Additionally, the finding that *Mcl-1* prevents rapamycin-induced autophagy suggests that *Mcl-1*-mediated inhibition of autophagy is downstream of mTOR. Various antiapoptotic proteins, including Bcl-2, Bcl-X_L_ Bcl-w, and, to a lesser extent, *Mcl-1*, interact with Beclin1, which prevents it from initiating autophagy [10,12,33,34]. Therefore, it is likely that *Mcl-1* inhibits the induction of autophagy during nutrient deprivation by suppressing activation of the Beclin-1-Vps34 PI3K complex.

Under nutrient-rich conditions, baseline autophagic activity is primarily involved in routine maintenance where it eliminates protein aggregates and defective organelles from the cellular environment. Our findings clearly show that *Mcl-1* did not interfere with the autophagy that is involved in cellular quality control. Instead, *Mcl-1* became a positive regulator of mitophagy, where it promoted the specific clearance of depolarized mitochondria in response to stress. Previous studies have reported functions for *Mcl-1* in mitophagy, but the findings are currently conflicting. For instance, Hollville et al. initially reported that antiapoptotic Bcl-2 members, including *Mcl-1*, inhibit the clearance of depolarized mitochondria by preventing Parkin’s translocation to mitochondria [16]. This study also performed coimmunoprecipitation experiments demonstrating that Bcl-X_L_ and *Mcl-1* interact with Parkin even under baseline conditions, thereby preventing its translocation to depolarized mitochondria, implicating *Mcl-1* in the early steps in the mitophagy pathway. However, Parkin and *Mcl-1* have distinct subcellular localizations at baseline where Parkin is localized to the cytosol, while *Mcl-1* is anchored to the outer mitochondrial membrane. Another limitation of this study is that the authors did not assess whether autophagosome formation was altered in cells overexpressing the various antiapoptotic Bcl-2 members, and it is therefore unclear if the reduction in mitophagy was linked to changes in autophagosome formation. A more recent study reported the BH3-only mimetic UMI-77 induces mitophagy by disrupting the interaction between *Mcl-1* and Bax/Bak [17]. This group also discovered that *Mcl-1* functions as a mitophagy receptor where it interacts with both LC3A and GABARAP on the autophagosome via an LIR motif to promote the clearance of mitochondria [17]. Here, we confirmed that *Mcl-1* can function as a mitophagy receptor to clear depolarized mitochondria in a Parkin-independent manner. However, our findings also suggest that this is not the sole mechanism by which *Mcl-1* promotes mitophagy. Mutating all three putative LIR motifs in *Mcl-1* did not completely abrogate mitophagy. Instead, our data suggest that *Mcl-1* might also be facilitating mitophagy by interacting with the mitophagy receptor Bnip3. Given the importance of clearing damaged mitochondria in response to stress, it seems likely that *Mcl-1* would have the capacity to enable this process in multiple ways. Bnip3 is an atypical BH3-only protein that is involved in regulating both cell death and mitophagy in cells. As a prodeath protein, Bnip3 induces cell death via Bax/Bak [35] and by promoting opening of the mitochondrial permeability transition pore [31]. Bnip3 also contains an LIR and promotes mitophagy by binding to LC3 on the autophagosome [19]. However, how Bnip3 switches between these two distinct functions is currently unclear. Our results suggest that *Mcl-1* might be involved in regulating the pro-mitophagy function of Bnip3. 

Finally, in contrast to the other Bcl-2 members, *Mcl-1* has a very short half-life and is rapidly degraded in response to apoptotic stimuli, which leads to activation of cell death [36]. It has also been reported that degradation of *Mcl-1* during nutrient deprivation is directly connected to the activation of autophagy [12]. Similarly, deletion of *Mcl-1* in neurons or in mice alone also leads to activation of autophagy. Although these findings suggest that degradation of *Mcl-1* leads to activation of autophagy, our discovery that autophagy was strongly induced in the infarct border zone despite unchanged levels of *Mcl-1* suggests that it does not have to be degraded for autophagy to proceed in response to stress. Similarly, overexpression of *Mcl-1* did not affect FCCP-induced activation of autophagy. Instead, it is possible that a post-translational modification (PTM) of *Mcl-1* functions to release its inhibition of autophagy and potentially changes its function towards being a pro-mitophagy protein. *Mcl-1* is subjected to a variety of PTMs, ranging from phosphorylation to SUMOylation, that alter its function and stability [36,37]. Future studies should focus on identifying the PTMs involved in regulating its functions in autophagy and mitophagy. 

To sum, *Mcl-1* is a key regulator of mitochondrial homeostasis where it modulates diverse, albeit interconnected, aspects of mitochondrial function, including mitochondrial permeability (apoptosis), mitochondrial morphology (fission), and turnover (mitophagy). Consequently, it is not surprising that deletion of *Mcl-1* leads to rapid loss of myocytes and cardiac dysfunction [15,18]. Heart failure is often associated with an accumulation of dysfunctional mitochondria and the death of myocytes, and therapeutic targeting of *Mcl-1* has already shown promise in other disease contexts [17,38,39,40]. Therefore, pharmacological activation of *Mcl-1* in the heart could induce mitophagy to protect myocytes from cell death activation and prevent cardiovascular disease development.

## Data Availability

The data that support the findings of this study are available from the corresponding author upon reasonable request.
